# Identification of a new variant of TTR involved in familial amyloid cardiomyopathy (FAC) in Brazil: from the patient to the protein

**DOI:** 10.1186/1750-1172-10-S1-P55

**Published:** 2015-11-02

**Authors:** Priscila Ferreira, Carolina Andrade, Ricardo Santanna, Natalia Varejão, Salvado Ventura, Fernando Palhano, Marcia Cruz, Debora Foguel

**Affiliations:** 1 Universidade Federal do Rio de Janeiro, Medical Biochemistry, 21941902, Rio de Janeiro, Brazil; 2 Universitat Autònoma de Barcelona, Bioquímica i Biologia Molecular, Barcelona, Spain; 3 Hospital Universitário Clementino Fraga Filho, Neurologia, Rio de Janeiro, Brazil

## Background

In Brazil, the most prevalent cases of TTR-related amyloidoses is the V30M variant due the Portuguese colonization. Our group has stablished a center for molecular diagnostic of FAP. Since then, we have sequenced almost one hundred patients form the University Hospital and their relatives. Recently, we identified a patient with a severe cardiomyopathy. This patient has a German ancestry and the sequence of his TTR gene revealed the presence of a new mutation, namely A19D. This patient presented heart failure and was classified by the NIHA as IV. We have also identified a patient, 66-years old, from a family with African ancestry, which bears the typical V122I mutation. This patient presented carpal tunnel syndrome and two years later developed heart failure that progressed to NYHA III. The main goal of the present work is to characterize the Brazilian population with FAC by combining bioinformatics and biophysical studies.

## Methods

We built a model for A19D by using FoldX (http://foldx.crg.es/) with the original WT-TTR structure as deposited in the PDB under code 1F41. The toxicity of amyloid aggregates composed of A19D and V122I were evaluated by using cell viability assay in primary culture of murine cardiomyocytes and fibroblasts as well as N2a cell line. Results: Initially we used the bioinformatics tool FoldX to predict the thermodynamic stability of the new mutant A19D. Our predictions have shown that the insertion of mutation caused a decrease in the thermodynamic stability of the protein and cause an electrostatic clash in the region of thyroxine channel that could facilitate their dissociation. A19D was purified heterologously and biophysical studies demonstrated that this mutant is a dimer and not a tetramer as wild type structure. The crystallographic structure of A19D is identical of wild type TTR. Thermodynamic studies with A19D indicated that it has a lower stability than the wild-type protein and other mutants. This new mutant has a faster aggregation kinetics forming amyloid fibers in two hours as shown by images. Amyloid aggregates of A19D and V122I were incubated with primary culture of cardiomyocytes and fibroblasts from murine heart and also in N2a cell line. The viability assay showed that the oligomers of A19D and V122I are toxic for cardiomyocytes and neuroblastoma cells and interestingly fibroblasts also suffer injury in the presence of these aggregates.

## Conclusions

The recent consolidation of TTR diagnosis in our University Hospital led to the identification of a rare, new variant of TTR in Brazil, namely, A19D, as well as the common V122I variant. A19D presented a marginal thermodynamic stability as inferred by bioinformatics and by biophysical studies with the purified protein. A19D showed to be dimer in solution. The viability assay shows that toxic mechanism displayed by this new mutant can be directly correlated with the aggressiveness observed in the disease developed by the patient.

